# Hip arthroscopy for femoroacetabular impingement is associated with significant improvement in early patient reported outcomes: analysis of 4963 cases from the UK non-arthroplasty registry (NAHR) dataset

**DOI:** 10.1007/s00167-022-07042-y

**Published:** 2022-07-14

**Authors:** Richard Holleyman, Mark Andrew Sohatee, Stephen Lyman, Ajay Malviya, Vikas Khanduja, NAHR User Group, Marcus J. K. Bankes, Tony Andrade, Tim Board, Jonathon Conroy, Matthew Wilson, Callum McBryde

**Affiliations:** 1 Population Health Sciences Institute, Newcastle University, Newcastle upon Tyne, England; 2 Northumbria NHS Foundation Trust, Newcastle upon Tyne, England; 3 The Hospital for Special Surgery, New York, NY USA; 4 Addenbrooke’s - Cambridge University Hospitals NHS Foundation Trust, Cambridge, England

**Keywords:** Hip, Hip arthroscopy, Impingement, Femoroacetabular impingement, Hip surgery, Cohort, Registry, Outcomes

## Abstract

**Purpose:**

Results from recent randomised controlled trials demonstrate the superiority of surgery over physiotherapy in patients with femoroacetabular impingement (FAI) of the hip in early follow-up. However, there is paucity of evidence regarding which factors influence outcomes of FAI surgery, particularly notable is the lack of information on the effect of impingement subtype (cam or pincer or mixed) on patient reported outcomes measures (PROMs). This study aims to evaluate the early outcomes of hip arthroscopy for FAI, and their determinants.

**Methods:**

This is a retrospective analysis of prospectively collected data from the UK Non-Arthroplasty Hip Registry (NAHR) of patients undergoing arthroscopic intervention for FAI between 2012 and 2019. The null hypothesis was that there is no difference in PROMs, based on morphological subtype of FAI treated or patient characteristics, at each follow-up timepoint. The outcome measures used for the study were the iHOT-12 score and the EQ5D Index and VAS 6- and 12-month follow-up.

**Results:**

A cohort of 4963 patients who underwent arthroscopic treatment of FAI were identified on the NAHR database. For all FAI pathology groups, there was significant improvement from pre-operative PROMs when compared to those at 6 and 12 months. Overall, two-thirds of patients achieved the minimum clinically important difference (MCID), and almost half achieved substantial clinical benefit (SCB) for iHOT-12 by 12 months. Pre-operatively, and at 12-month follow-up, iHOT-12 scores were significantly poorer in the pincer group compared to the cam and mixed pathology groups (*p* < 0.01). Multivariable analysis revealed PROMS improvement in the setting of a higher-grade cartilage lesion.

**Conclusion:**

This registry study demonstrates that hip arthroscopy is an effective surgical treatment for patients with symptomatic FAI and results in a statistically significant improvement in PROMs which are maintained through 12 months follow-up.

**Level of evidence:**

III.

**Supplementary Information:**

The online version contains supplementary material available at 10.1007/s00167-022-07042-y.

## Introduction

Femoroacetabular impingement (FAI) can give rise to hip pain, reduced range of movement, loss of function, and eventually arthritis of the hip [22, 26]. This condition can be classified as one of three morphological types; cam, pincer, or mixed [13]. The primary aim of surgical treatment of FAI is hip preservation through decreasing abnormal contact stresses across the hip joint, which can subsequently delay progression to osteoarthritis [50] and potentially improve patients’ symptoms and quality of life. Whilst Burman described direct visualisation of the hip joint through arthroscopic means in a cadaveric study in 1931 [4], Ganz originally described FAI and also *‘open surgical dislocation’* for surgical management of FAI [12]. However, arthroscopic management of FAI has now become standard of care despite initial scepticism [2, 3, 16, 36, 45].

Results from randomised control trials are now available which demonstrate the superiority of surgery over physiotherapy in patients with FAI [15, 34]. There are a number of studies that have discussed patient reported outcomes for FAI surgery, some of these have been small [5–11, 14, 19, 21, 25, 29, 30, 32, 35, 39, 40, 48, 51, 53–59, 61] however, the Danish Hip Arthroscopy Registry (DHAR), like the UK NAHR, have been able to demonstrate the outcomes of large numbers of patients undergoing surgery for FAI [43, 44]. Efficacy has also been demonstrated in adolescents as well as young adults [41]. Aside from the publications from the DHAR, there is also a paucity of studies looking at differences in patient reported outcomes measure (PROMs) based on morphology type, in FAI and the corresponding improvements in PROMs scores.

The aim of this study, therefore, was to [1] report the demographics and early outcomes for patients undergoing hip arthroscopy for FAI up to 12 months follow-up and [2] to compare outcomes by FAI pathology groups using data from the UK NAHR. The null hypothesis was that there would be no difference in terms of the 12 months iHOT-12 gain vs pre-operative scores when comparing FAI pathology groups.

## Materials and methods

**Fig. 1:**
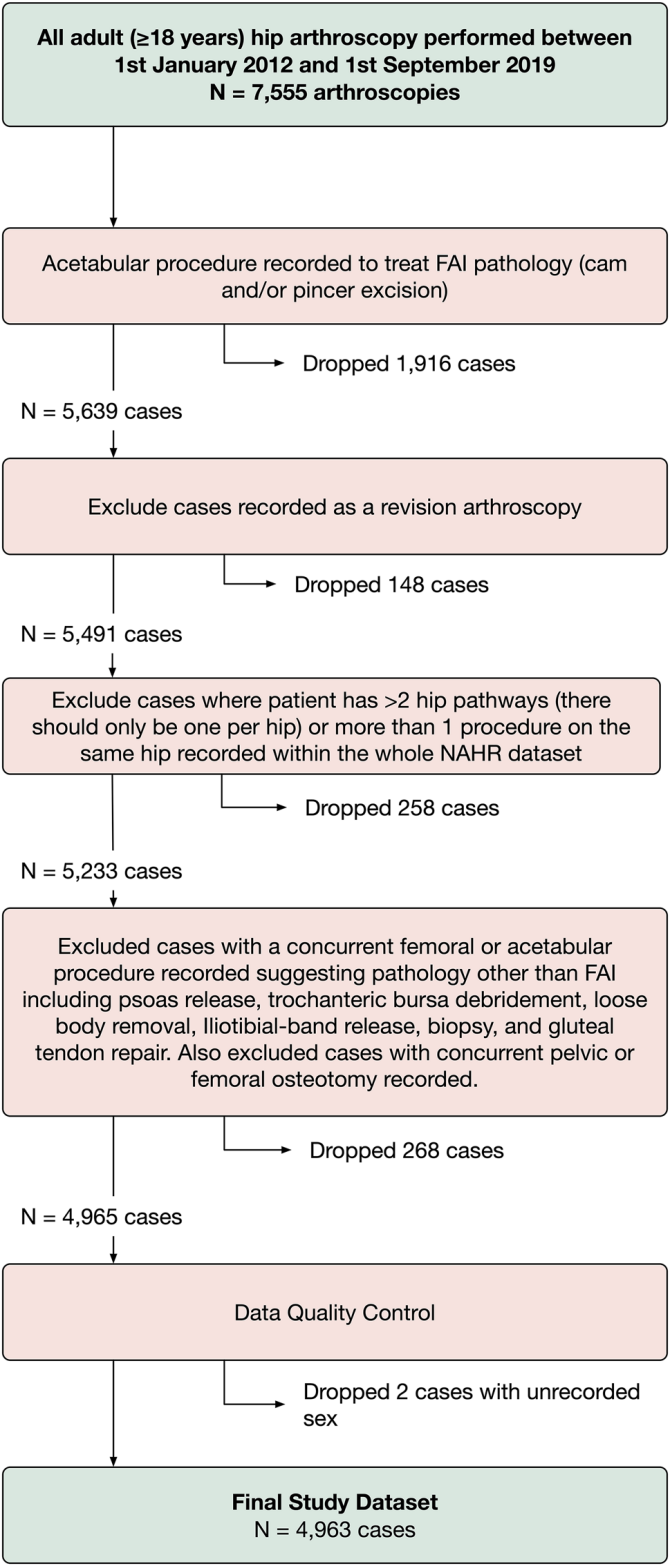
Study flow diagram

Approval for this observational study was granted by the NAHR steering committee (reference NAHR/2018/02). All patients 14 years and older who underwent a hip arthroscopy procedure to address an FAI lesion between 2012 and 2019 were identified from the NAHR database (Fig. 1, study flow diagram). Procedures used to define FAI were excision of femoral “cam” lesion and/or excision of acetabular “pincer” lesion (including rim recession [simple], rim recession with labral reattachment, and sub-spinous resection).

Patient demographics and data related to clinical diagnosis and surgical procedure are collected and uploaded by trained staff using a structured electronic form which allows for recording of isolated or simultaneous femoral and acetabular procedures. Patients who consent to data collection receive questionnaires to determine EQ-5D Index and iHOT-12 scores pre-operatively and at six months, one, two- and five-year follow-up. Follow-up data was captured in September 2020 to allow a minimum of 12 months follow-up. Due to the low return-rate of patient questionnaires at two or more years follow-up in this cohort (<5% of cases returned 2-year PROMS) it was only possible to report outcomes up to one year post-operatively. Body mass index (BMI) was also collected and reported as both a continuous and a categorical variable according to World Health Organisation (WHO) groups. Where available, acetabular chondral damage was determined as the single worst area of cartilage damage recorded intra-operatively according to Konan et al.’s [28] description (Grade 1—wave sign with intact chondrolabral junction, Grade 2—Chondrolabral junction separation but no delamination, Grade 3—Delamination, Grade 4—Exposed bone) [20]. Femoral cartilage defects were classified according to the Outerbridge classification [52].

Patients were classified according to pathology into three groups dependent upon the index procedure performed for FAI which comprised: (1) “cam”: excision of cam lesion (with no recorded procedure to treat pincer pathology during the same surgery), (2) “pincer”: excision pincer lesion (with no recorded procedure to treat cam pathology during the same surgery) and (3) “mixed”: excision of both a cam and pincer lesion during the same operation. Concurrent femoral and acetabular procedures performed in association with the index procedure which defined the pathology group were also determined for each pathology group (Table 1).

**Table 1:** Concurrent femoral and acetabular procedures recorded for the study cohort by index FAI pathology treated

	FAI Pathology Group					
	Cam		Pincer		Mixed	
	Procedure	*n* (%)	Procedure	*n* (%)	Procedure	*n* (%)
Femoral Procedures Recorded	Cam removal^a^	2971 (100%)	Femoral Osteophyte Removal	36 (7.8%)	Cam removal^a^	1530 (100%)
	Femoral Osteophyte Removal	36 (1.2%)	Other Femoral Procedure	16 (3.5%)	Femoral Osteophyte Removal	61 (4%)
	Femoral Cartilage Debridement	31 (1%)	Femoral Cartilage Debridement	10 (2.2%)	Femoral Cartilage Debridement	29 (1.9%)
	Femoral Microfracture	24 (0.8%)	Femoral Microfracture	9 (1.9%)	Femoral Microfracture	14 (0.9%)
	Other Femoral Procedure	14 (0.5%)	Chondral Treatment	1 (0.2%)	Other Femoral Procedure	7 (0.5%)
	Femoral Graft/ACI	1 (0%)				
Acetabular Procedures Recorded	Acetabular Cartilage Debridement	1167 (39.3%)	Acetabular Rim Recession (Labral Re-Attachment)^a^	229 (49.6%)	Acetabular Rim Recession (Simple)^a^	764 (49.9%)
	Acetabular Labral Debridement	1149 (38.7%)	Acetabular Rim Recession (Simple)^a^	223 (48.3%)	Acetabular Rim Recession (Labral Re-Attachment)^a^	721 (47.1%)
	Acetabular Labral Repair	1144 (38.5%)	Acetabular Labral Debridement	153 (33.1%)	Acetabular Labral Repair	687 (44.9%)
	Acetabular Microfracture	254 (8.5%)	Acetabular Labral Repair	151 (32.7%)	Acetabular Labral Debridement	439 (28.7%)
	Acetabular Labral Resection	117 (3.9%)	Acetabular Cartilage Debridement	57 (12.3%)	Acetabular Cartilage Debridement	383 (25%)
	Other Acetabular Procedure	104 (3.5%)	Acetabular Labral Resection	40 (8.7%)	Other Acetabular Procedure	152 (9.9%)
	Acetabular Cartilage Reattachment	40 (1.3%)	Other Acetabular Procedure	29 (6.3%)	Subspinous Resection^a^	149 (9.7%)
	Acetabular Graft/ACI	32 (1.1%)	Subspinous Resection^a^	26 (5.6%)	Acetabular Labral Resection	134 (8.8%)
	Acetabular Pincer Removal	2 (0.1%)	Acetabular Cartilage Reattachment	16 (3.5%)	Acetabular Microfracture	97 (6.3%)
			Acetabular Microfracture	15 (3.2%)	Acetabular Cartilage Reattachment	30 (2%)
			Acetabular Graft/ACI	3 (0.6%)	Acetabular Graft/ACI	6 (0.4%)

^a^Denotes procedures used to define pathology groups. N.B. Multiple procedures may be performed on the same patient

Several thresholds for the minimum clinically important difference (MCID) and substantial clinical benefit (SCB) following hip arthroscopy for FAI have been reported in the literature [23]. For patients in whom pre and post-operative outcome scores were recorded at 12 month follow-up, an improvement in iHOT-12 score of ≥13 and ≥28 were used as reference criteria for achieving MCID and SCB respectively as reported by Martin et al. [37] and Kivlan et al.[27] Additionally, the proportion of cases achieving an increase in iHOT-12 score greater than or equal to thresholds from 1 to 40 were calculated, in order that the results can be compared with the existing and future literature and provide evidence to support informed patient consent.

### Statistical analysis

Bivariate analyses were conducted using Chi-square tests for categorial outcomes and Student’s *t* test or ANOVA as appropriate for continuous outcomes. BMI data were missing in 40% of cases; to maximise the number of observations available for multivariable modelling BMI was therefore treated as a categorical variable and these patients assigned to a “missing” group. Statistical analysis was performed in STATA (StataCorp. 2017. Stata Statistical Software: Release 15. College Station, TX: StataCorp LLC.) and R (R Core Team [2019]. R: A language and environment for statistical computing, Vienna, Austria).

## Results

A series of 4963 arthroscopies performed for treatment of FAI were identified from the NAHR database. A significantly higher proportion of female patients were observed in the pincer group (Table 2; *p* < 0.0001). Overall, for the entire cohort, there was significant improvement in pre-operative scores which were maintained up to 12 months (Table 3).

**Table 2:** Study cohort demographics by FAI pathology group

Variable	Cam	Pincer	Mixed	Overall	*p* value
No. of cases (%)	2971 (59.9%)	462 (9.3%)	1530 (30.8%)	4963 (100%)	
Mean Age (SD) (years)	35.8 (10.8)	35.2 (10.4)	35.3 (10.4)	35.6 (10.6)	0.326 (ANOVA)
Sex [no. (%)]					<0.0001 (Chi-Squared)
Female	1575 (53.0%)	343 (74.2%)	782 (51.1%)	2700 (54.4%)	
Male	1396 (47.0%)	119 (25.8%)	748 (48.9%)	2263 (45.6%)	
Mean BMI (SD) (kg/m^2^)	25.5 (4.6); *n* = 1847 (62.2%)	25.8 (5.1); *n* = 216 (46.8%)	25.8 (4.5); *n* = 918 (60.0%)	25.7 (4.6); *n* = 2981 (60.1%)	0.280 (ANOVA)
BMI Group [no. (%)]					< .0001 (Chi-Squared)
<25 kg/m^2^	933 (31.4%)	114 (24.7%)	434 (28.4%)	1481 (29.8%)	
25–30 kg/m^2^	630 (21.2%)	61 (13.2%)	344 (22.5%)	1035 (20.9%)	
≥30 kg/m^2^	284 (9.6%)	41 (8.9%)	140 (9.2%)	465 (9.4%)	
Missing	1124 (37.8%)	246 (53.2%)	612 (40.0%)	1982 (39.9%)	
Severity of single worst zone of acetabular chondral damage^a^ [no. (%)]					< .0001 (Chi-Squared)
No Chondral Damage	329 (11.1%)	44 (9.5%)	133 (8.7%)	506 (10.2%)	
Grade 1: Wave Sign with intact chondrolabral junction	492 (16.6%)	82 (17.7%)	209 (13.7%)	783 (15.8%)	
Grade 2: Chondrolabral junction separation but no delamination	509 (17.1%)	56 (12.1%)	295 (19.3%)	860 (17.3%)	
Grade 3: Cartilage Delamination	566 (19.1%)	72 (15.6%)	340 (22.2%)	978 (19.7%)	
Grade 4: Exposed bone	310 (10.4%)	15 (3.2%)	130 (8.5%)	455 (9.2%)	
Not Recorded	765 (25.7%)	193 (41.8%)	423 (27.6%)	1381 (27.8%)	
Femoral Outerbridge [no. (%)]					< 0.0001 (Chi-Squared)
No Chondral Damage	1425 (48.0%)	154 (33.3%)	568 (37.1%)	2147 (43.3%)	
Grade 1: Rough Surface, Chondral Softening	241 (8.1%)	49 (10.6%)	237 (15.5%)	527 (10.6%)	
Grade 2: Irregular Surface defects < 50% Cartilage Depth	244 (8.2%)	18 (3.9%)	122 (8.0%)	384 (7.7%)	
Grade 3: > 50% Loss of Cartilage Depth	119 (4.0%)	15 (3.2%)	66 (4.3%)	200 (4.0%)	
Grade 4: Full Thickness Loss	54 (1.8%)	7 (1.5%)	25 (1.6%)	86 (1.7%)	
Not Recorded	888 (29.9%)	219 (47.4%)	512 (33.5%)	1619 (32.6%)	

^a^Single worst area of chondral damage across all acetabular zones [20, 28]

**Table 3:** Outcome scores by FAI pathology group

Variable	FAI pathology group				
	Cam	Pincer	Mixed	Overall	*p* value
Mean outcome score^a^					
Pre-op iHOT-12	33.1 (32.4–33.8); *n* = 2569 (86.5%)	28.8 (26.9–30.7);* n* = 315 (68.2%)	32.9 (31.9–33.9);* n* = 1286 (84.1%)	32.7 (32.2–33.3); *n* = 4170 (84.0%)	< 0.001
Pre-op EQ-5D Index	0.52 (0.52–0.53); *n* = 2648 (89.1%)	0.49 (0.46–0.51); *n* = 360 (77.9%)	0.52 (0.51–0.53);* n* = 1319 (86.2%)	0.52 (0.51–0.53);* n* = 4327 (87.2%)	0.014
Pre-op EQ-5D VAS	66.7 (65.9–67.5); *n* = 2656 (89.4%)	66.1 (63.9–68.3);* n* = 361 (78.1%)	66.5 (65.4–67.7);* n* = 1321 (86.3%)	66.6 (65.9–67.2);* n* = 4338 (87.4%)	0.886
6-mo iHOT-12	59.1 (57.7–60.5); *n* = 1390 (46.8%)	53.4 (49.6–57.2);* n* = 195 (42.2%)	57.3 (55.3–59.3);* n* = 723 (47.3%)	58.0 (56.9–59.1); *n* = 2308 (46.5%)	0.015
6-mo EQ-5D Index	0.67 (0.66–0.68); *n* = 1459 (49.1%)	0.65 (0.61–0.68);* n* = 232 (50.2%)	0.66 (0.64–0.68);* n* = 766 (50.1%)	0.67 (0.66–0.67);* n* = 2457 (49.5%)	0.181
6-mo EQ-5D VAS	69.6 (68.4–70.7); *n* = 1462 (49.2%)	67.9 (64.9–71.0);* n* = 232 (50.2%)	69.5 (67.9–71.1);* n* = 767 (50.1%)	69.4 (68.5–70.3); *n* = 2461 (49.6%)	0.587
12-mo iHOT-12	61.0 (59.4–62.6); *n* = 1219 (41.0%)	53.5 (49.4–57.5);* n* = 182 (39.4%)	58.1 (56.0–60.2);* n* = 685 (44.8%)	59.4 (58.2–60.6);* n* = 2086 (42.0%)	< 0.01
12-mo EQ-5D Index	0.69 (0.68–0.70); *n* = 1266 (42.6%)	0.65 (0.61–0.68); *n* = 191 (41.3%)	0.67 (0.65–0.69);* n* = 718 (46.9%)	0.68 (0.67–0.69);* n* = 2175 (43.8%)	0.047
12-mo EQ-5D VAS	71.4 (70.2–72.7); *n* = 1272 (42.8%)	67.2 (63.7–70.7);* n* = 192 (41.6%)	69.8 (68.1–71.5); *n* = 718 (46.9%)	70.5 (69.5–71.5);* n* = 2182 (44.0%)	0.040
At 6 months					
Change vs. pre-op.^b^					
Change iHOT-12—6-mo	+ 26.5 (25.0–27.9);* n* = 1223 (41.2%); *p* ≤ 0.0001	+ 23.8 (19.4–28.2);* n* = 148 (32.0%); *p* ≤ 0.0001	+ 26.2 (24.1–28.3);* n* = 618 (40.4%); *p* ≤ 0.0001	+ 26.2 (25.0–27.3); *n* = 1989 (40.1%); *p* ≤ 0.0001	0.507
Change EQ-5D Index—6-mo	+ 0.15 (0.14–0.16); * n* = 1319 (44.4%); *p* ≤ 0.0001	+ 0.15 (0.11–0.19); * n* = 191 (41.3%); *p* ≤ 0.0001	+ 0.14 (0.12–0.16); * n* = 670 (43.8%); *p* ≤ 0.0001	+ 0.15 (0.14–0.16); *n* = 2180 (43.9%); *p* ≤ 0.0001	0.830
Change EQ-5D VAS—6-mo	+ 3.3 (1.9–4.7);* n* = 1323 (44.5%); *p* ≤ 0.0001	+ 1.7 (−1.8 to 5.3); * n* = 191 (41.3%); *p* = 0.336	+ 3.5 (1.6–5.4); *n* = 671 (43.9%); *p* ≤ 0.001	+ 3.2 (2.1–4.3); *n* = 2185 (44.0%); *p* ≤ 0.0001	0.693
% Achieving MCID iHOT-12 at 6-mo	Yes = 832 of 1223 (68.0%) / No = 391 of 1223 (32.0%)	Yes = 98 of 148 (66.2%) / No = 50 of 148 (33.8%)	Yes = 413 of 618 (66.8%) / No = 205 of 618 (33.2%)	Yes = 1343 of 1989 (67.5%) / No = 646 of 1989 (32.5%)	0.821
% Achieving SCB iHOT-12 at 6-mo	Yes = 567 of 1223 (46.4%) / No = 656 of 1223 (53.6%)	Yes = 66 of 148 (44.6%) / No = 82 of 148 (55.4%)	Yes = 291 of 618 (47.1%) / No = 327 of 618 (52.9%)	Yes = 924 of 1989 (46.5%) / No = 1065 of 1989 (53.5%)	0.857
At 12 months					
Change vs. pre-op.^b^					
Change iHOT-12—12-mo	+ 27.0 (25.4–28.6); * n* = 1101 (37.1%); *p* ≤ 0.0001	+ 23.9 (19.5–28.3); * n* = 150 (32.5%); *p* ≤ 0.0001	+ 25.4 (23.3–27.6); * n* = 603 (39.4%); *p* ≤ 0.0001	+ 26.2 (25.0–27.5); * n* = 1854 (37.4%); *p* ≤ 0.0001	0.291
Change EQ-5D Index—12-mo	+ 0.16 (0.14–0.17); * n* = 1177 (39.6%); *p* ≤ 0.0001	+ 0.15 (0.11–0.20); * n* = 164 (35.5%); *p* ≤ 0.0001	+ 0.14 (0.12–0.16); * n* = 648 (42.4%); *p* ≤ 0.0001	+ 0.15 (0.14–0.16); * n* = 1989 (40.1%); *p* ≤ 0.0001	0.353
Change EQ-5D VAS—12-mo	+ 4.4 (2.9–5.8); * n* = 1186 (39.9%); *p* ≤ 0.0001	+ 1.3 (−3.2 to 5.7); * n* = 165 (35.7%); *p* = 0.573	+ 2.8 (0.8–4.9); * n* = 649 (42.4%); *p* ≤ 0.01	+ 3.6 (2.5–4.8); * n* = 2000 (40.3%); *p* ≤ 0.0001	0.243
% Achieving MCID iHOT-12 at 12-mo	Yes = 741 of 1101 (67.3%) / No = 360 of 1101 (32.7%)	Yes = 94 of 150 (62.7%) / No = 56 of 150 (37.3%)	Yes = 397 of 603 (65.8%) / No = 206 of 603 (34.2%)	Yes = 1232 of 1854 (66.5%) / No = 622 of 1854 (33.5%)	0.491
% Achieving SCB iHOT-12 at 12-mo	Yes = 527 of 1101 (47.9%) / No = 574 of 1101 (52.1%)	Yes = 62 of 150 (41.3%) / No = 88 of 150 (58.7%)	Yes = 280 of 603 (46.4%) / No = 323 of 603 (53.6%)	Yes = 869 of 1854 (46.9%) / No = 985 of 1854 (53.1%)	0.312

^a^The values are given as the mean score (95%CI); number (%) of cases available for follow-up

^b^For cases with pre and post-operative follow-up data, the values are given as the mean score improvement (95%CI); number (%) of cases available for follow-up; *p* value derived with the paired *t* test

Pre-operatively, and at 6, and 12-month follow-up, iHOT-12 scores (unadjusted) were significantly poorer in the pincer group compared to the cam and mixed pathology groups (*p* < 0.01) with a similar relationship observed for EQ-5D Index scores. Within each pathology group, patients experienced statistically significant improvement in baseline pre-operative iHOT-12 and EQ-5D Index scores at 6 months which was maintained up to 12 months post-operatively (all *p* < 0.0001), however there were no significant between group differences in the magnitude of iHOT-12 or EQ-5D Index of VAS score improvement on univariable analysis.

### Outcome scores by age group

**Fig. 2:**
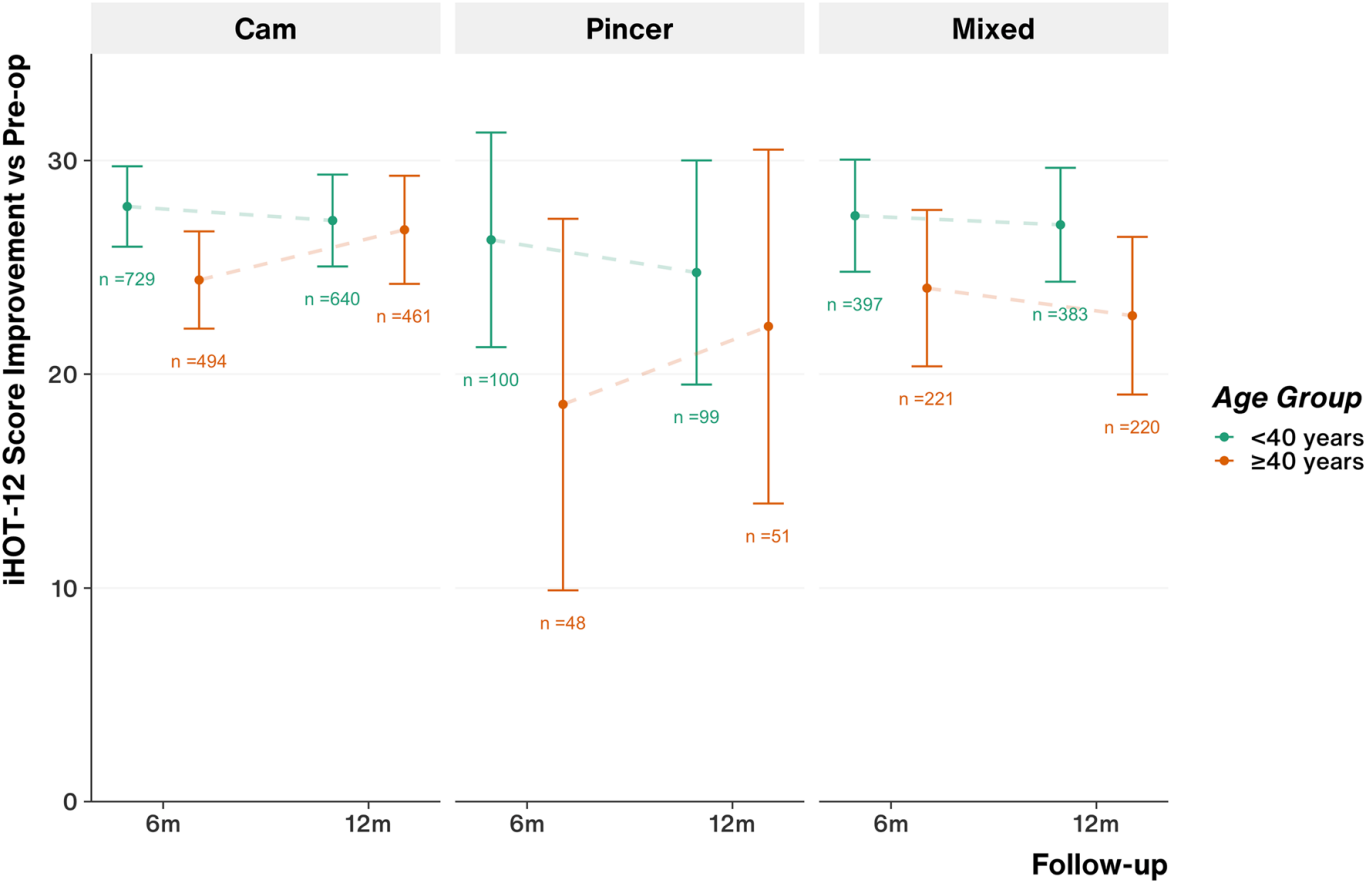
iHOT-12 Score improvement (compared to pre-operative baseline) for each FAI pathology group comparing patients under and over 40 years. Point and error bar represents the mean and 95% confidence interval. Annotation represents the number of cases for each data point

For all FAI pathology types, significant improvement in iHOT-12 score was maintained up to 12 months post-operatively for younger (under 40 years) and older age groups. Older patients with isolated cam pathology started from significantly higher baseline iHOT-12 scores but had significantly poorer iHOT-12 improvement by 6 months compared to younger patients, however, statistical significance was lost by 12 months (Fig. 2). At 12 months, there were no differences in 12-month EQ-5D Index scores or degree of improvement for all FAI pathology groups when comparing younger and older patients and all maintained significant improvement compared to pre-operative scores.

### Outcome scores by sex

**Fig. 3:**
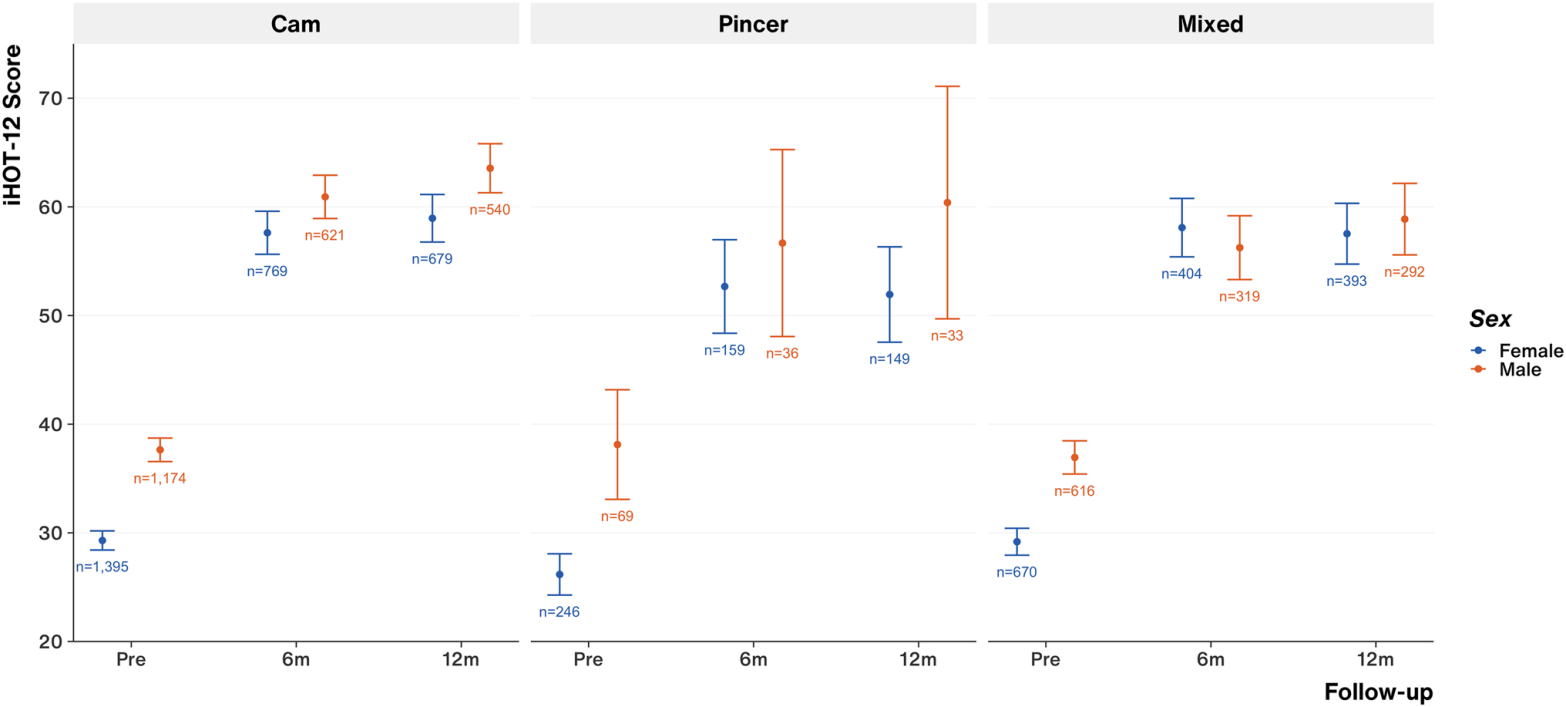
Pre- and post-operative (6-month and 12-month) patient iHOT-12 score by FAI pathology group and patient gender. Point and error bar represents the mean and 95% confidence interval. Annotation represents the number of cases for each data point

Considering all FAI pathology groups combined, male patients started from a significantly higher pre-operative baseline iHOT-12 and reached significantly higher iHOT-12 scores by 12 months compared to female patients; this significance was largely driven by better post-operative outcome scores in male patients who underwent treatment for cam pathology only (Fig. 3).

For each FAI pathology group and when considering all FAI pathology groups collectively, both male and female patients saw significant improvement (delta) in their baseline pre-operative iHOT-12 score up to 6- and 12-months following surgery (all *p* < 0.01).

### MCID & SCB

**Fig. 4:**
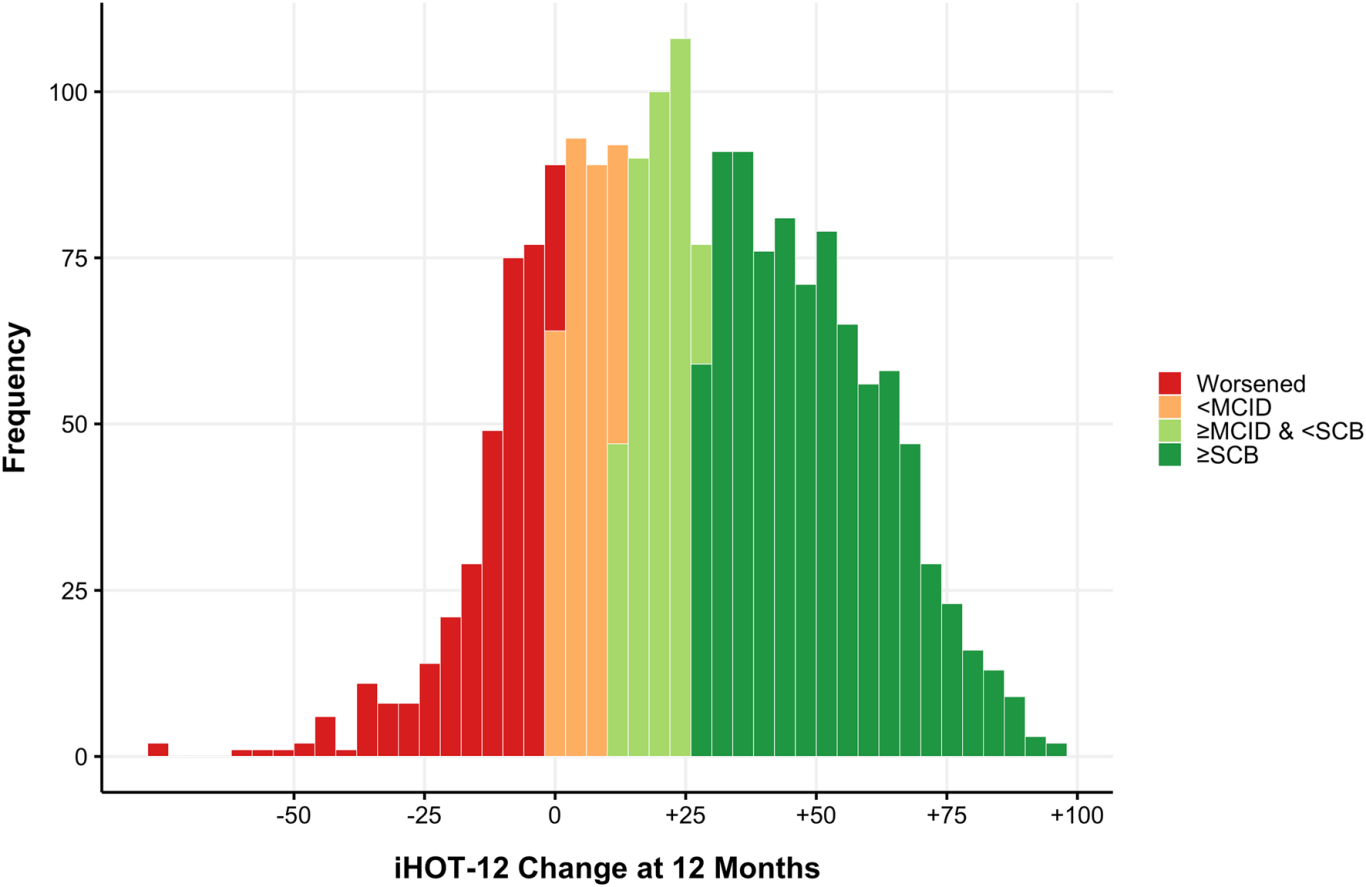
Histogram showing the distribution of iHOT-12 score improvement (delta) at 12 months vs pre-operatively. Bars are highlighted to illustrate cases achieving MCID (iHOT-12 improvement ≥ 13) and SCB (iHOT-12 improvement ≥ 28). Bin width = 4 points

Overall, 67% of patients achieved the MCID (increase in iHOT-12 of ≥13) at up to 12 months (Fig. 4). The proportion of cases achieving MCID in the pincer group (63%) was lower than in cam (67%) and mixed (66%) pathology groups (Fig. 3). Overall, 47% of patients achieved SCB (increase in iHOT-12 of ≥28) by 12 months. Again, the proportion of cases achieving SCB in the pincer group (41%) was lower than in cam (48%) and mixed (46%) pathology groups. Data showing the proportion of cases achieving iHOT-12 improvement for thresholds between +1 and +40 are available in the supplementary materials (Supplementary material Table 1).

### Predictors of PROMS improvement

Considering the overall cohort including all FAI pathology groups, patients who returned both pre and 12-month post-operative iHOT-12 questionnaires were, on average, older (36.6 versus 35.0 years, *p* < 0.0001) and more likely to be female (*p* < 0.0001) than those who did not return one or both questionnaires (*p* < 0.0001, supplementary table 2); there were also differences in the proportions of femoroacetabular impingement (FAI) pathology seen (*p* = 0.022). Differences in 12-month follow-up rates between the FAI pathology groups (Cam = 37.1%, Pincer = 32.5% and Mixed = 39.4%) may, therefore, confound the primary outcome measure of iHOT-12 improvement (delta) as responders are inherently different to non-responders. To attempt to account for the above, a combination of random sampling and propensity score matching were used to create a synthetic cohort of cases for each FAI pathology group to balance the demographic differences that may have arisen from differences in follow-up rates between the three groups. This methodology is described fully in the supplementary materials and adapted from methods published previously by this group [18]. This synthetic cohort (*n* = 1000, supplementary materials Table 3) was then used as the basis of a multivariable linear regression model predicting iHOT-12 12-month improvement.

Age, sex, severity of any recorded acetabular chondral lesion, BMI, pre-operative outcome score and FAI pathology group were used to predict iHOT-12 score improvement at 12 months in multivariable analysis after the creation of a novel synthetic cohort (*n* = 1000, patient characteristics are reported in supplementary table 3). Whilst not statistically significant, pincer pathology and a higher-grade chondral lesion showed the largest association with poorer iHOT-12 improvement at 12 months, however, there was evidence of a linear trend with respect to increasing chondral damage (Table 4). Chondral grading was therefore re-modelled as an ordered categorical variable (after excluding 341 [34%] patients in the synthetic cohort with missing chondral grading data) which confirmed a statistically significant association between increasing lesion grade and detrimental iHOT-12 improvement (coefficient −1.94 (95%CI −3.56, −0.31, *p* = 0.020).

**Table 4:** Multivariable model

Variable	Multivariable Predictors of iHOT-12 Improvement at 12-months (Co-efficient, 95%CI, *p* value)
FAI Group	
Cam	Reference
Pincer	−3.9 (−9.9 to 2.1), 0.201
Mixed	−1.1 (−4.6 to 2.5), 0.548
Age	
<40 years	Reference
≥40 years	−2.7 (−6.5 to 1.0), 0.153
Sex	
Female	Reference
Male	0.9 (−2.6 to 4.3), 0.627
BMI Group	
<25	Reference
25–30	−0.6 (−6.0 to 4.7), 0.821
≥ 30	−2.5 (−8.6 to 3.5), 0.410
Missing	−0.1 (−4.4 to 4.2), 0.959
Acetabular Chondral Damage (Severity of single worst zone)	
No Chondral Damage	Reference
Grade 1: Wave Sign with intact chondrolabral junction	0.2 (−6.3 to 6.7), 0.946
Grade 2: Chondrolabral junction separation but no delamination	2.5 (−4.4 to 9.4), 0.476
Grade 3: Cartilage Delamination	−3.0 (−9.5 to 3.5), 0.362
Grade 4: Exposed bone	−7.3 (−15.2 to 0.6), 0.070
Not Recorded	−3.3 (−9.3 to 2.6), 0.274
Pre-op iHOT-12 Score	−0.4 (−0.5 to −0.3), 0.000
*N*	1000
r2	0.1

## Discussion

The principal finding of this study was that hip arthroscopy is an effective surgical treatment for patients with symptomatic FAI and was associated with significant improvement in patient outcomes which are maintained at 12 months follow-up. MCID is achieved in two-thirds of patients and just under half of patients have a substantial clinical benefit by 12 months. Outcomes appear better for cam and mixed pathologies as compared with pincer type lesions.

This study demonstrates that the majority of patients, irrespective of age, demonstrated a statistically significant improvement in outcome scores, although patients under 40 years may obtain greater benefit. When looking at previously published studies, age is a recognised determining factor for outcome. Domb et al. show, age to be a predictive factor for post-operative Non Arthritic Hip Score (NAHS) [9] and Kaldau et al. found that age significantly influenced the Hip Sports Activity Scale (HSAS) and, furthermore, they identified that there is a statistically significant difference in post-operative HSAS when comparing patients less than or equal to 40 years of age versus those over 40. This is mirrored in a study by McCormick et al. who stated that undergoing surgery for FAI at 39 years of age or younger was predictive of good to excellent results at a minimum of 2 years after surgery compared with a patient 40 years or older [38]. Likewise Lin et al. demonstrated that middle aged (35–50 years) and older patients (51–75 years) experienced greater declines in outcomes, when compared to young patients (15–34) [31].

When considering sex, males had significantly higher pre-operative baseline PROMs, as well as higher post-operative PROMs at 12 months. As a result, females have a significantly greater improvement in PROMs at 12 months. Indeed, the studies published in the literature which looked at outcomes based on sex [11, 17, 57] only consider the final PROM score with results matching this study’s findings. In these, the absolute score was statistically significantly better for males; however, this does not consider the degree of clinical improvement patients see, which is important to consider when selecting patients for operative intervention. Joseph et al. also considered the change in PROM score, as well as absolute values, and their findings echoed this study, with greater change/improvement in scores seen in females at 6 and 12 months [24].

Saltzman et al. have also looked at the influence of BMI on outcomes in FAI surgery and identified significant differences in PROMs based on BMI. They observed normal BMI patients, followed by underweight patients, demonstrating greater scores than their overweight, obese, and morbidly obese counterparts [56]. In multivariable modelling, similar trends were observed in the present study regarding lower score improvement in obese patients as compared to patients of normal BMI, however this did not reach statistical significance.

Data on the effect of FAI morphology type (cam, pincer, mixed) on patient reported outcomes appears to be limited, particularly when considering such a large number of procedures, with the exception of published work from the DHAR [43]. Beck et al. identified that the morphology of FAI influences the pattern of damage seen in the hip but did not correlate this with PROMs data [1]. Moon et al. found no difference in PROMs (Harris Hip Score and WOMAC) scores based on morphological subtype [42]. In the present study, iHOT-12 scores pre-operatively and at 12 months were significantly worse in the pincer group, than those in the cam and mixed group. Palmer et al. noted that, when using the Harris Hip Score (HHS) patients with mixed cam and pincer deformity has worse outcomes than those with cam alone, however, they excluded patients with “pincer only” FAI and therefore comparison between all morphological types could not be made [53].

Nho et al. looked at survivorship and outcome of hip arthroscopy for FAI syndrome performed with modern surgical techniques and reported MCID achievement of 73% in a series of 935 patients [47]. Similarly, Nwachukwu et al. reported that 68.1% patients in a series of 364 patients achieved improvement beyond MCID for Hip Outcome Score—Activities of Daily Living [37, 49] and Malviya et al. found that 76.6% patients had an improvement in QoL, (Quality of Life) measured in improvement of QoL score[46], after arthroscopic surgery for FAI [33]. In this study, 67% of patients achieved improvement in their i-HOT12 scores beyond the MCID of 13, this was greater in patients with cam and mixed pathologies as compared with pincer. The overall figure seems to be slightly less than previous reports and this might be explained by the NAHR including data submitted by surgeons of differing levels of experience including low-volume surgeons, whilst all the published evidence reports are from high volume centres with experienced surgeons. This aspect of the study makes it pragmatic and allows for realistic deductions to be made.

The NAHR allows for the capture of national data, within the UK, pertaining to ‘non-arthroplasty’ elective hip surgery procedures. It has been demonstrated that database studies are better at reporting certain outcome measures, such as: complications, revision and conversion to arthroplasty, when compared with than conventional original research publications, outlining their importance [60]. Furthermore, registries allow collection of data from low-volume surgeons and those of different skill sets presenting a more realistic picture of achievable results than results from high volume surgeons in specialist centres. The present study includes results from 69 surgeons contributing between 1 and 555 cases (mean = 72 cases, median = 25 cases) and whilst outcomes were not adjusted for surgeon experience, results of this study show arthroscopy for FAI to be a procedure providing clinical benefit to patients amongst the breadth of surgeons contributing to the NAHR.

The authors note the limitations to this study, including largely the relatively short duration of follow-up. The NAHR collects scores pre-operatively and at six months, one, two- and five-year follow-up. Due to the low return-rate of patient outcome scores at two or more years follow-up, in this cohort it was only possible to report outcomes up to one year post-operatively. However, the authors also recognise that the NAHR will continue to evolve over time and could allow for the development of more robust data collection strategies moving forward. The lessons learned from it may also be useful in the development of other registries which would allow for further population-based studies to be undertaken. Other limitations include: no standardisation of the surgical decision making process amongst surgeons, lack of operative standardisation and post-operative standardisation between centres and that, nationally, not all surgeons are contributing to the NAHR. Furthermore, by not capturing radiographic parameters it is not possible to correlate the influence of these on PROM scores and assumes the categorisation of morphological subtype by operating surgeon was correct.

To the best of the authors’ knowledge, this is one of the largest studies to date, reporting short term patient reported outcomes of hip arthroscopy for the management of FAI in a pragmatic setting. The only study known to the authors that includes a larger number of procedures is that from the DHAR that looked at more than 5000 procedures [43].

## Conclusion

Arthroscopic treatment of symptomatic FAI was associated with statistically significant improvement in patient reported clinical outcome scores for the majority of patients, however, consideration to specific groups must be paid, such as; extremes of age, high BMI and the presence of pincer pathology, at least, in the short term. The outcomes may not be universally successful and this study provides important evidence to facilitate patient-clinician discussion regarding expected outcomes and guide informed consent.

## Supplementary Information

Below is the link to the electronic supplementary material.

Supplementary file1 (DOCX 424 KB)
