# Supplementary material for: Hip arthroscopy for femoroacetabular impingement is associated with significant improvement in early patient reported outcomes: analysis of 4963 cases from the UK non-arthroplasty registry (NAHR) dataset
Source: Knee Surg Sports Traumatol Arthrosc. 2022 Jul 14;31(1):58–69. doi: 10.1007/s00167-022-07042-y (PMC9859857; doi:10.1007/s00167-022-07042-y)
Supplement: Supplementary file 1 — Supplementary file1 (DOCX 424 KB) [file 167_2022_7042_MOESM1_ESM.docx]

Supplemental Materials

## Supplementary Table 1

Table showing the number and proportion of cases achieving each given threshold of iHOT-12 improvement vs. pre-operative baseline by FAI procedure group.

| Threshold for iHOT-12 gain vs pre-op at 12 months | Cam  (n = 1,101) | Pincer  (n = 150) | Mixed  (n = 603) | Overall  (n = 1,854) |
| --- | --- | --- | --- | --- |
| ≥1 | 899 (81.7%) | 119 (79.3%) | 485 (80.4%) | 1,503 (81.1%) |
| ≥2 | 888 (80.7%) | 118 (78.7%) | 476 (78.9%) | 1,482 (79.9%) |
| ≥3 | 877 (79.7%) | 118 (78.7%) | 464 (76.9%) | 1,459 (78.7%) |
| ≥4 | 857 (77.8%) | 115 (76.7%) | 460 (76.3%) | 1,432 (77.2%) |
| ≥5 | 843 (76.6%) | 113 (75.3%) | 456 (75.6%) | 1,412 (76.2%) |
| ≥6 | 834 (75.7%) | 111 (74.0%) | 449 (74.5%) | 1,394 (75.2%) |
| ≥7 | 816 (74.1%) | 109 (72.7%) | 441 (73.1%) | 1,366 (73.7%) |
| ≥8 | 798 (72.5%) | 107 (71.3%) | 432 (71.6%) | 1,337 (72.1%) |
| ≥9 | 785 (71.3%) | 106 (70.7%) | 427 (70.8%) | 1,318 (71.1%) |
| ≥10 | 774 (70.3%) | 104 (69.3%) | 418 (69.3%) | 1,296 (69.9%) |
| ≥11 | 763 (69.3%) | 102 (68.0%) | 410 (68.0%) | 1,275 (68.8%) |
| ≥12 | 751 (68.2%) | 97 (64.7%) | 403 (66.8%) | 1,251 (67.5%) |
| ≥13 | 741 (67.3%) | 94 (62.7%) | 397 (65.8%) | 1,232 (66.5%) |
| ≥14 | 729 (66.2%) | 89 (59.3%) | 389 (64.5%) | 1,207 (65.1%) |
| ≥15 | 716 (65.0%) | 86 (57.3%) | 383 (63.5%) | 1,185 (63.9%) |
| ≥16 | 704 (63.9%) | 85 (56.7%) | 377 (62.5%) | 1,166 (62.9%) |
| ≥17 | 690 (62.7%) | 83 (55.3%) | 370 (61.4%) | 1,143 (61.7%) |
| ≥18 | 673 (61.1%) | 80 (53.3%) | 363 (60.2%) | 1,116 (60.2%) |
| ≥19 | 661 (60.0%) | 78 (52.0%) | 355 (58.9%) | 1,094 (59.0%) |
| ≥20 | 645 (58.6%) | 76 (50.7%) | 346 (57.4%) | 1,067 (57.6%) |
| ≥21 | 631 (57.3%) | 74 (49.3%) | 337 (55.9%) | 1,042 (56.2%) |
| ≥22 | 618 (56.1%) | 72 (48.0%) | 329 (54.6%) | 1,019 (55.0%) |
| ≥23 | 601 (54.6%) | 69 (46.0%) | 324 (53.7%) | 994 (53.6%) |
| ≥24 | 585 (53.1%) | 69 (46.0%) | 316 (52.4%) | 970 (52.3%) |
| ≥25 | 563 (51.1%) | 69 (46.0%) | 310 (51.4%) | 942 (50.8%) |
| ≥26 | 553 (50.2%) | 65 (43.3%) | 298 (49.4%) | 916 (49.4%) |
| ≥27 | 535 (48.6%) | 62 (41.3%) | 290 (48.1%) | 887 (47.8%) |
| ≥28 | 527 (47.9%) | 62 (41.3%) | 280 (46.4%) | 869 (46.9%) |
| ≥29 | 514 (46.7%) | 61 (40.7%) | 275 (45.6%) | 850 (45.8%) |
| ≥30 | 505 (45.9%) | 59 (39.3%) | 268 (44.4%) | 832 (44.9%) |
| ≥31 | 495 (45.0%) | 59 (39.3%) | 256 (42.5%) | 810 (43.7%) |
| ≥32 | 480 (43.6%) | 58 (38.7%) | 247 (41.0%) | 785 (42.3%) |
| ≥33 | 471 (42.8%) | 55 (36.7%) | 241 (40.0%) | 767 (41.4%) |
| ≥34 | 449 (40.8%) | 53 (35.3%) | 237 (39.3%) | 739 (39.9%) |
| ≥35 | 437 (39.7%) | 50 (33.3%) | 231 (38.3%) | 718 (38.7%) |
| ≥36 | 423 (38.4%) | 45 (30.0%) | 223 (37.0%) | 691 (37.3%) |
| ≥37 | 412 (37.4%) | 44 (29.3%) | 218 (36.2%) | 674 (36.4%) |
| ≥38 | 398 (36.1%) | 44 (29.3%) | 209 (34.7%) | 651 (35.1%) |
| ≥39 | 385 (35.0%) | 41 (27.3%) | 202 (33.5%) | 628 (33.9%) |
| ≥40 | 376 (34.2%) | 40 (26.7%) | 191 (31.7%) | 607 (32.7%) |

# Multivariable Analysis Methodology

Considering the overall cohort including all FAI pathology groups, we found that patients who returned ***both*** pre ***and*** 12-month post-operative iHOT-12 questionnaires were, on average, older (36.6 versus 35.0 years, p < 0.0001), more likely to be female (p < 0.0001), and more likely to be missing data for body mass index than those who did not return questionnaires (p<0.0001). There were also differences in the proportions of femoroacetabular impingement (FAI) pathology groups (p = 0.022, supplementary table 2).

Differences in 12-month follow-up rates between the FAI pathology groups (Cam = 37.1%, Pincer = 32.5% and Mixed = 39.4% follow-up rates for proportion of patients returning pre and post-operative scores) may therefore confound our primary outcome measure of iHOT-12 gain as responders are inherently different to non-responders. Thus, we developed a novel methodology to attempt to create a synthetic cohort of cases in each FAI pathology group to balance the demographic differences that may have arisen from differences in follow-up rates between the three groups.

## Supplementary Table 2

Key patient and surgical characteristics for the entire cohort stratified by those who returned pre and 12-months post-operative iHOT-12 questionnaires (responders) and those who did not (non-responders).

| Variable | Non-Responder | Responder | Overall | p value |
| --- | --- | --- | --- | --- |
| No. of cases (%) | 3,109 (62.6%) | 1,854 (37.4%) | 4,963 (100%) |  |
| Mean Age (SD) (yr) | 35.0 (10.7) | 36.6 (10.4) | 35.6 (10.6) | <0.0001**** (t-test) |
| Sex [no. (%)] |  |  |  | <0.0001**** (Chi-Squared) |
| Female | 1,610 (51.8%) | 1,090 (58.8%) | 2,700 (54.4%) |  |
| Male | 1,499 (48.2%) | 764 (41.2%) | 2,263 (45.6%) |  |
| Mean BMI (SD) (kg/m2) | 25.7 (4.6); n=1,737 (55.9%) | 25.6 (4.5); n=1,244 (67.1%) | 25.7 (4.6); n=2,981 (60.1%) | 0.320 (t-test) |
| BMI Group [no. (%)] |  |  |  | <0.0001**** (Chi-Squared) |
| <25 | 851 (27.4%) | 630 (34.0%) | 1,481 (29.8%) |  |
| 25-30 | 595 (19.1%) | 440 (23.7%) | 1,035 (20.9%) |  |
| ≥30 | 291 (9.4%) | 174 (9.4%) | 465 (9.4%) |  |
| Missing | 1,372 (44.1%) | 610 (32.9%) | 1,982 (39.9%) |  |
| FAI Type [no. (%)] |  |  |  | 0.022* (Chi-Squared) |
| Cam | 1,870 (60.1%) | 1,101 (59.4%) | 2,971 (59.9%) |  |
| Pincer | 312 (10.0%) | 150 (8.1%) | 462 (9.3%) |  |
| Mixed | 927 (29.8%) | 603 (32.5%) | 1,530 (30.8%) |  |

## Methodology

We used a combination of random sampling and propensity score matching in order to attempt to account for the impact of a the above, building upon a similar methodology we have applied in previous work. [1]

1.

Patients were classified according to whether they did (‘responders’) or did not (‘non-responders’) return iHOT-12 questionnaires both pre-operatively and at 12 months post-operatively.

2.

We randomly sampled 20% of cases from the 2,971 cases in the Cam group (drawing from all responders and non-responders). This random sample of 20% cases would, on average, be expected to select 220 responders (37.1% [the Cam iHOT-12 responder rate] multiplied by the random sample size (20% of 2,971 = 594.2) = 220 responders).

3.

Propensity score matching (1 to 1 matching without replacement, with a random match order and including the following co-variates – age, sex, BMI and severity of worst acetabular chondral lesion) was then used to match these 594 randomly selected patients to their closest match in the group of Cam ‘responders’ - with matches being allowed to be drawn from the entire sample of Cam responders (n = 1,101). The expectation was that the 220 randomly selected responders would match back to themselves and the remaining randomly selected non-responders (on average 374 cases: 594 minus 220 responders) would each match to one of the remaining responders available to match and with the closest propensity score (i.e. one of the 1,101 responder cases which had not already matched to a responder).

This yields a dataset of 594 matched responders. The propensity score matching step allows the creation of a synthetic cohort of responder patients who are demographically more similar to the original cohort comprising both responders and non-responders.

4.

Steps 2-3 were repeated over 1,000 iterations.

5.

The resultant 1,000 matched responder datasets of 594 patients each were then combined and for each unique responder we counted the number of times the case had been sampled over the 1,000 iterations. On average, over 1,000 iterations, we would expect each unique Cam responder case to have been selected 594 ÷ 1,101 × 1000 = 540 times (95% binomial confidence interval 509.5 to 569.3) which is confirmed in supplementary figure A.

6.

In order to select only those responders who were most similar to the overall cohort of responders and non-responders, we excluded those responders who were likely to be less representative of the overall cohort on the basis that they were selected fewer times than the lower 95% binomial confidence interval of the probability of selection if the process were completely random.

We therefore selected only those Cam responders who were selected more than 509.5 times which yielded 588 cases.

7.

Steps 2-6 were then repeated for the Pincer and Mixed FAI pathology groups sampling 20% of cases for each iteration.

The number of selections of each Pincer and Mixed pathology group case over 1,000 iterations is shown in supplementary figure B & C. According to the same calculation described in step 6 we only included Pincer and Mixed group responders who were selected more than 530 or 466 times respectively. This yielded 83 and 329 cases in the Pincer and Mixed groups respectively.

8.

The resultant cases for each FAI pathology group identified through steps 1 to 7 were then combined to create a synthetic cohort of 1,000 patients who were therefore the responders who were most representative of the overall cohort of responders and non-responders (supplementary table 3).

This synthetic cohort was then used as the basis of a linear regression model predicting iHOT-12 12-month improvement (model results are presented in table 4 in the main manuscript).

## Supplementary Figure A

Number of times each unique responder in the Cam pathology group was included in the randomly selected responder cohort after 1,000 iterations.


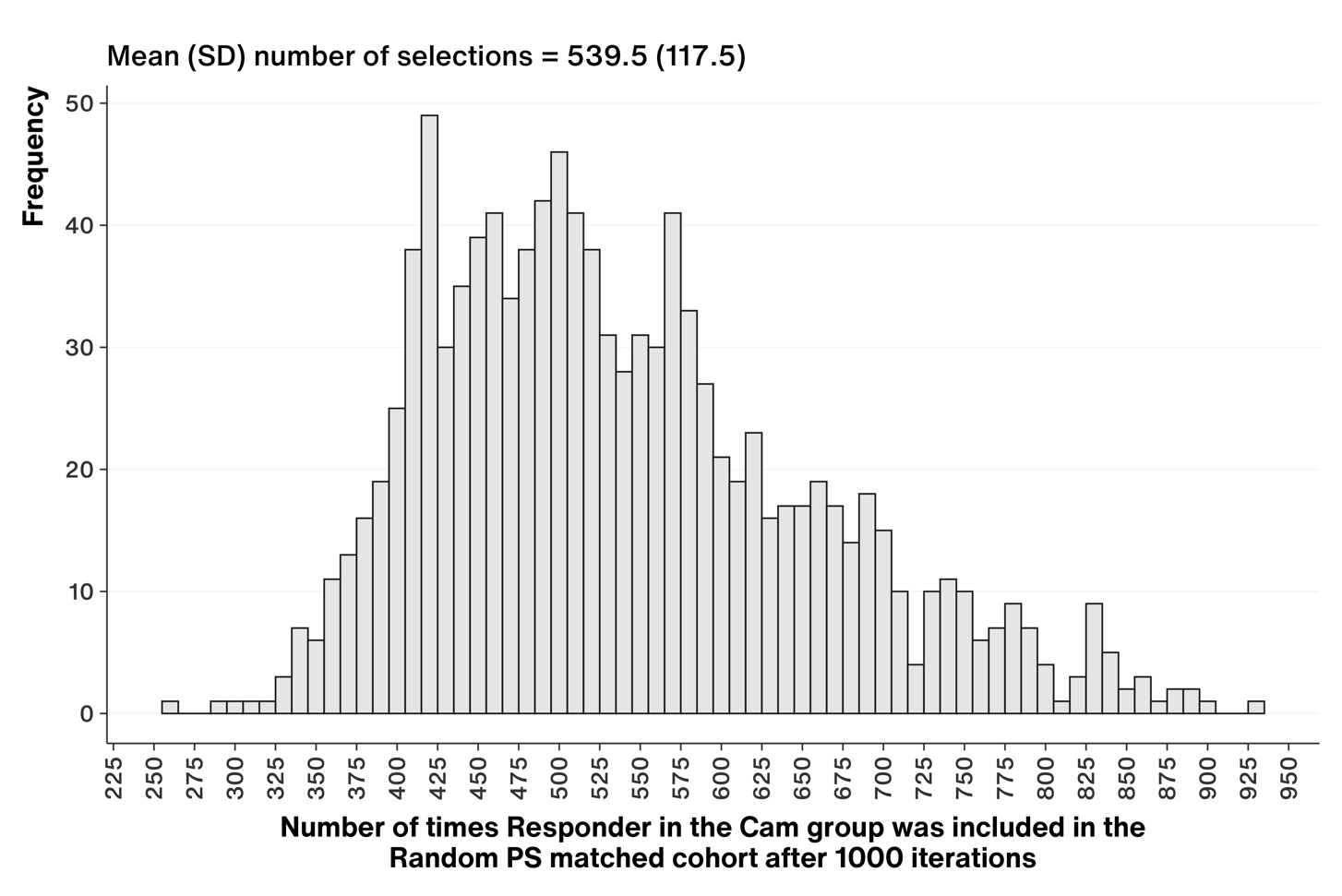


## Supplementary Figure B

Number of times each unique responder in the Pincer FAI pathology group was included in the randomly selected responder cohort after 1,000 iterations.


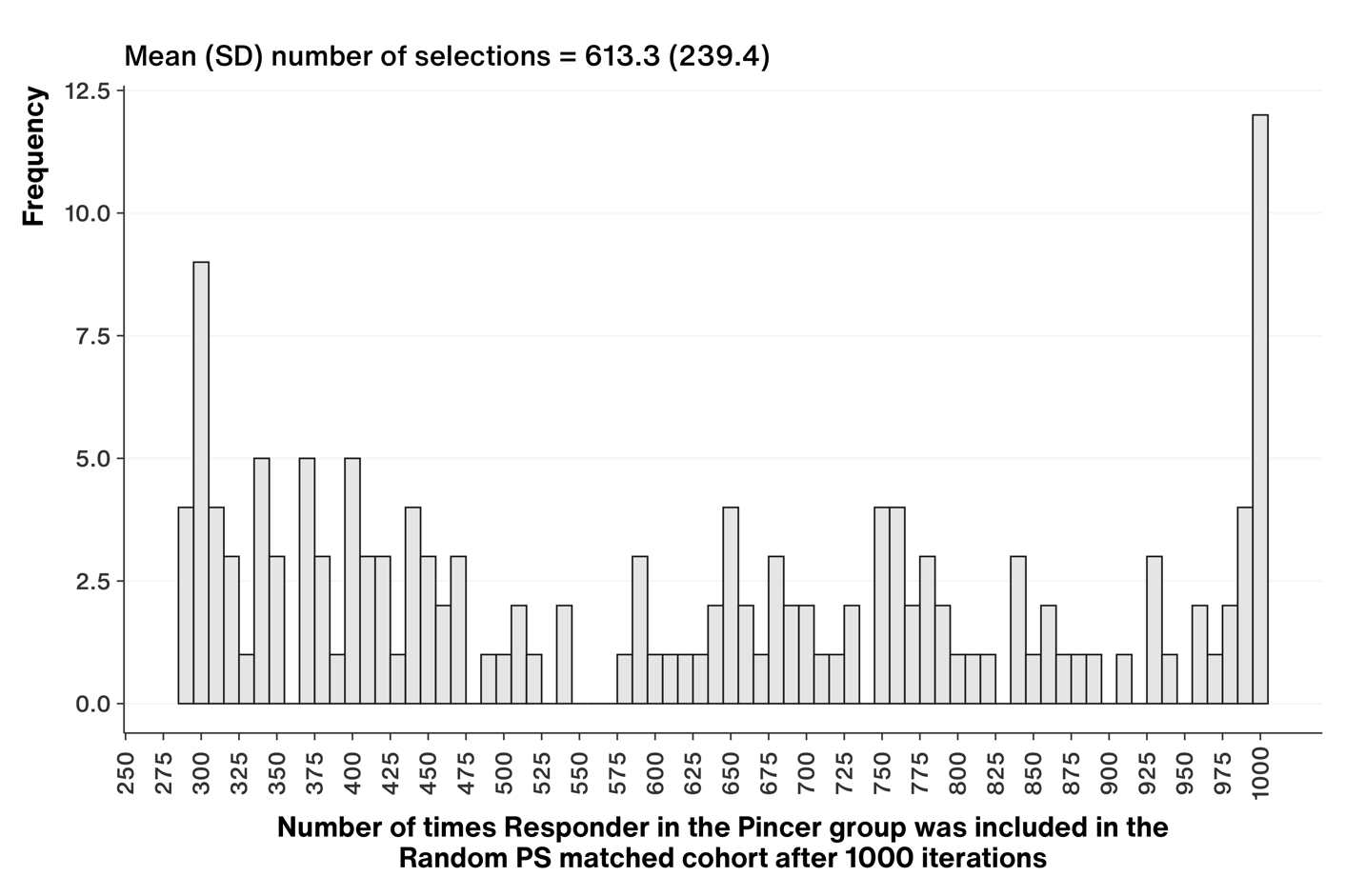


##
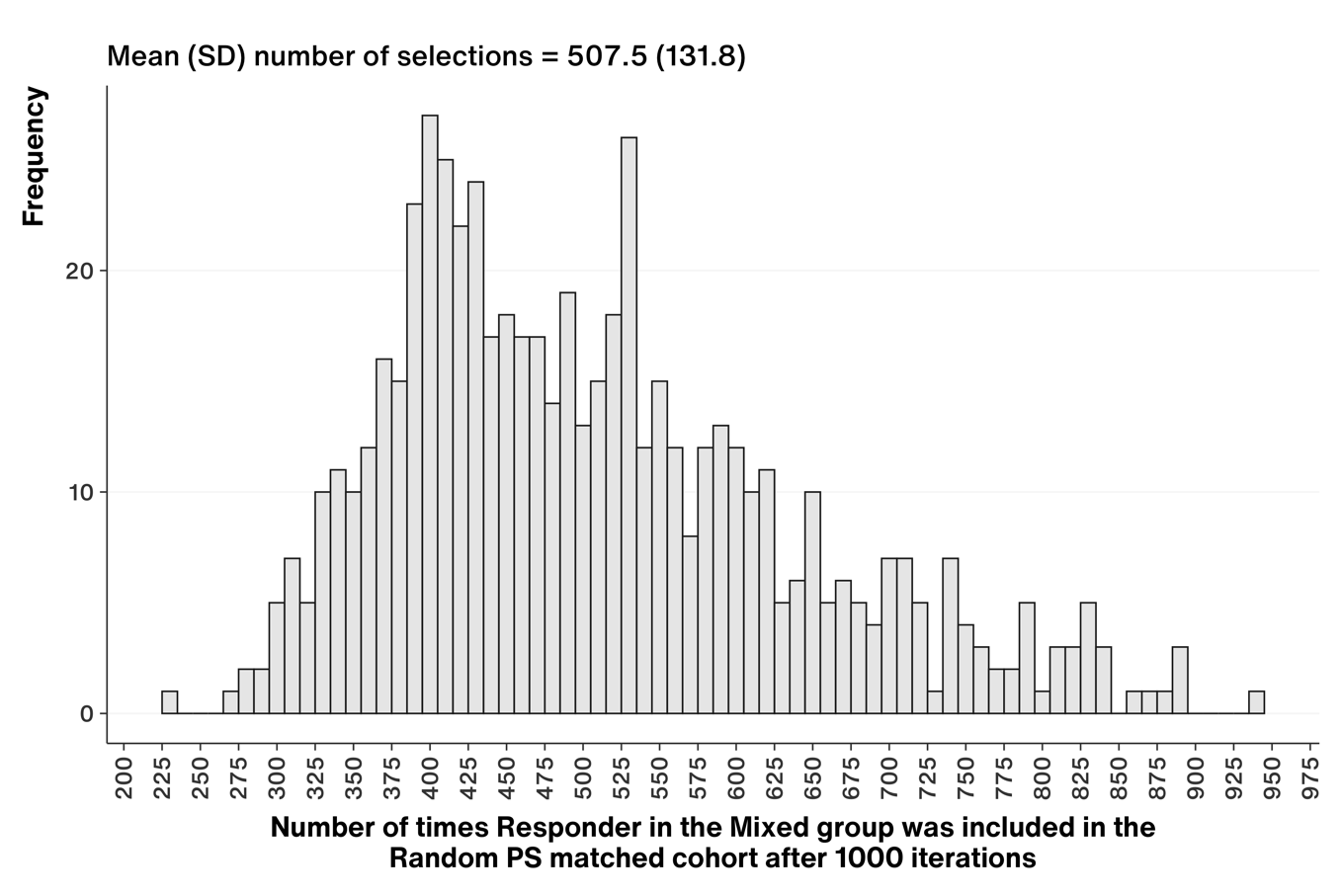
Supplementary Figure C

Number of times each unique responder in Mixed FAI pathology group was included in the randomly selected responder cohort after 1,000 iterations.

## Supplementary Table 3

Patient characteristics of synthetic cohort.

|  | FAI Procedure | | |  |
| --- | --- | --- | --- | --- |
| Variable | Cam | Pincer | Mixed | Total |
| n (%) | 588 (58.8%) | 83 (8.3%) | 329 (32.9%) | 1,000 (100%) |
| Mean age (SD) | 32.7 (9.3) | 37.3 (11.3) | 34.8 (9.8) | 33.8 (9.7) |
| Age (years) |  |  |  |  |
| <40 years | 446 (75.9%) | 50 (60.2%) | 225 (68.4%) | 721 (72.1%) |
| ≥40 years | 142 (24.1%) | 33 (39.8%) | 104 (31.6%) | 279 (27.9%) |
| Sex |  |  |  |  |
| Female | 283 (48.1%) | 59 (71.1%) | 134 (40.7%) | 476 (47.6%) |
| Male | 305 (51.9%) | 24 (28.9%) | 195 (59.3%) | 524 (52.4%) |
| BMI group |  |  |  |  |
| <25 | 123 (20.9%) | 13 (15.7%) | 72 (21.9%) | 208 (20.8%) |
| 25-30 | 99 (16.8%) | 9 (10.8%) | 50 (15.2%) | 158 (15.8%) |
| ≥30 | 78 (13.3%) | 6 (7.2%) | 28 (8.5%) | 112 (11.2%) |
| Missing | 288 (49.0%) | 55 (66.3%) | 179 (54.4%) | 522 (52.2%) |
| Severity of single worst zone of acetabular chondral damage* |  |  |  |  |
| No Chondral Damage | 55 (9.4%) | 4 (4.8%) | 36 (10.9%) | 95 (9.5%) |
| Grade 1 - Wave Sign with intact chondrolabral junction | 125 (21.3%) | 16 (19.3%) | 32 (9.7%) | 173 (17.3%) |
| Grade 2 - Chondrolabral junction separation but no delamination | 61 (10.4%) | 14 (16.9%) | 55 (16.7%) | 130 (13.0%) |
| Grade 3 - Cartilage Delamination | 97 (16.5%) | 11 (13.3%) | 75 (22.8%) | 183 (18.3%) |
| Grade 4 - Exposed bone | 58 (9.9%) | 1 (1.2%) | 19 (5.8%) | 78 (7.8%) |
| Not Recorded | 192 (32.7%) | 37 (44.6%) | 112 (34.0%) | 341 (34.1%) |
| Mean pre-op iHOT-12 score (SD) | 33.3 (18.4) | 29.5 (18.6) | 33.1 (17.7) | 32.9 (18.2) |

# Supplementary Material References

1. Holleyman R, Sohatee MA, Witt J, et al. Periacetabular Osteotomy for Developmental Dysplasia of the Hip and Femoroacetabular Impingement: A Study Using the U.K. Non-Arthroplasty Hip Registry (NAHR) Data Set. JBJS 2020;**102**(15):1312-20 doi: 10.2106/jbjs.18.01387published Online First: Epub Date]|.
